# Establishing a certificate in the analysis of medical data: a cross-sectional evaluation of a continuing professional development course in biostatistics in for healthcare professionals in Qatar

**DOI:** 10.1186/s12909-025-07999-7

**Published:** 2025-10-17

**Authors:** Saima Ali, Deema Al-Sheikhly, Laudy Mattar, Phyllis Sui Muffuh Navti, Soha Roger Dargham, Padraig Mark Healy, Ziyad Riyad Mahfoud

**Affiliations:** 1 Division of Continuing Professional Development, Weill Cornell Medicine- Qatar, Doha, Qatar; 2 Division of Medical Education, Weill Cornell Medicine – Qatar, Doha, Qatar

**Keywords:** Biostatistics education, Continuing professional development (CPD), Research capacity-building, Healthcare professionals

## Abstract

**Background:**

Healthcare professionals often face challenges in conducting and publishing research, citing a lack of biostatistical knowledge and skills as a significant barrier. In response, a Continuing Professional Development (CPD) course was developed to enhance local research capacity.

**Objective:**

This study aimed to evaluate the perceived impact of the biostatistics CPD course on self-reported knowledge, competence, and research engagement among healthcare professionals in Qatar.

**Method:**

A cross-sectional descriptive analysis was conducted using routine data collected in accordance with CPD accreditation requirements. Data included attendance records, certificate completions, and findings from two self-report surveys administered post-course. The surveys assessed perceived changes in knowledge and competence, achievement of course objectives, barriers to certificate completion, and long-term outcomes such as manuscript development and publication.

**Results:**

The majority of participants (88%) reported improved knowledge, 86% reported increased competence, and 54% credited the course with supporting their ability to draft a manuscript. Challenges were more frequently noted in advanced topics such as survival analysis and regression modeling.

**Conclusion:**

The CPD course in biostatistics yielded self-reported improvements in knowledge, competence, and performance among healthcare professionals. Challenges in advanced topics and certificate completion suggest the need for extended course duration, cost-effective online platforms, and enduring materials. Future studies using longitudinal designs and inferential methods could provide deeper insights into the long-term impact of such training initiatives.

**Supplementary Information:**

The online version contains supplementary material available at 10.1186/s12909-025-07999-7.

## Introduction

Empirical research plays a pivotal role in healthcare and the advancement of scientific and medical knowledge. To practice evidence-based medicine effectively, healthcare professionals (HCPs) need skills to comprehensively assess original research, staying informed of the latest findings. This entails critically evaluating the study’s design, data analysis, and interpretation of findings to make informed clinical decisions [1]. Furthermore, HCPs are encouraged to engage in research, with evidence suggesting benefits to patient care outcomes, healthcare performance, and workforce satisfaction [2–4].

The increasing complexity of medical data and the growing reliance on evidence-based medicine require HCPs worldwide to have a solid foundation in biostatistics. However, studies have consistently shown that many HCPs struggle with statistical literacy, leading to difficulties in data interpretation, critical appraisal of research, and implementation of research findings in clinical practice [5]. Addressing these gaps through structured training programs is crucial to ensure that HCPs can effectively contribute to research and apply statistical reasoning in their work.

Priorities for research can vary between countries due to differences in disease epidemiology and healthcare systems. Consequently, localized data plays a crucial role in the development of novel treatments or alternative approaches to ensure relevant and optimal service provision [5–7].

Qatar has prioritized research capacity-building as part of its Qatar National Vision 2030 (QNV 2030), aiming to transform into a knowledge-based economy with education, innovation, and healthcare at its core [8]. These priorities are consistent with regional and global strategies, including the Word Health Organization’s (WHO) call to strengthen health research systems and the United Nations Sustainable Development Goals promoting lifelong learning [9–11]. Strengthening biostatistical skills is central to these efforts, enabling HCPs to contribute to high-quality research and evidence-based care [5, 6, 11].

However, research conducted among HCPs in various countries worldwide consistently highlights deficiencies in understanding research methodology, particularly in statistical methods as prominent impediments to research progress [9, 12–14]. The Middle East faces additional challenges in biostatistics education, including a lack of dedicated postgraduate training programs, variability in statistical curricula within medical schools, and a reliance on international resources that may not be contextually relevant [10, 11]. Additionally, research culture in the region is still developing, with many HCPs citing insufficient institutional support and mentorship as barriers to advancing their research skills [5, 10]. These findings are echoed in the Middle East, with several studies revealing poor levels of knowledge in biostatistics among different HCPs, particularly regarding statistical software and advanced statistical methods, such as survival analysis and regression modeling [5, 7, 9, 15–18].

Within the Qatari context, research endeavors have primarily centered on pharmacists, with studies indicating a positive inclination among them to actively participate in all facets of the research process including the analysis and interpretation of data [5, 19]. Despite their confidence in the research process, many acknowledge difficulties in statistical analyses using software like IBM Statistical Package for the Social Sciences (IBM SPSS) and Stata, as well as in applying inferential statistical tests [5].

A lack of proficiency in biostatistics therefore limits the ability of HCPs to conduct and disseminate high quality research [6, 7]. Moreover, these findings bear implications beyond academic concerns, affecting the reliability of research outcomes and undermining evidence-based decision-making in healthcare [20]. Thus, a foundational understanding of biostatistics is critical for the effective implementation of evidence-based practice in healthcare [21].

The limited availability of postgraduate statistics courses designed for busy HCPs presents a challenge for those aiming to enhance their statistical proficiency amidst demanding work schedules [9]. In response a Continuing Professional Development (CPD) course in biostatistics tailored to the needs of HCPs was established. The course was strategically designed as an accredited continuing education (CE)/CPD activity to address the inherent challenges faced by HCPs who face barriers to participation due to time constraints. Acknowledging the mandatory nature of professional development for HCPs, the course would allow a structured and accredited avenue for professionals to enhance their proficiency in biostatistics. The accreditation requirements underscored the need to collect data and information to evaluate whether the course aims and objectives have been met through a CPD activity. Following the data collection, CPD providers are tasked with reviewing the information, comparing it to anticipated changes, and making necessary adjustments for future courses to enhance effectiveness. This systematic approach facilitates a process of quality improvement for accredited CPD providers.

Despite the recognized importance of biostatistics training, limited research exists on the effectiveness of CPD programs in this area, particularly for HCPs in Qatar. Few studies have assessed whether such training initiatives translate into improved research engagement, competence, and productivity. This study addresses this gap by evaluating a CPD biostatistics course tailored to the needs of HCPs in Qatar.

This paper aims to describe the development of a biostatistics course for HCPs and present participant feedback derived from the evaluation process following two cohorts of HCPs completing the training. The evaluation data focuses on participants’ self-reported achievement of course objectives, post-CPD activity and perceived impacts on three criteria: knowledge, competence, and performance. The analysis also identifies potential barriers and challenges in completing the certificate of analysis and meeting course objectives.

## Method

### Process for the development of the certificate

The establishment and delivery of the program was a multistage process designed to conform to the Division of Healthcare Professionals and Accreditation Council for Continuing Medical Education’s (ACCME) standards [22, 23]. The process began with a gap analysis informed by a review of the literature and the personal experiences of the course director-an experienced senior statistician actively engaged in advising HCPs on statistical analysis and study design. The next phase of the process advanced with the collaboration of the CPD division at Weill Cornell Medicine-Qatar. This engagement was essential to ensure that the course development adhered to accreditation requirements and that measurable learning outcomes were identified.

To align with accreditation standards, a scientifically diverse advisory committee was convened to deliberate on key aspects such as course content, software selection, and the practical applicability of the curriculum within their respective fields. Through this collaboration a three-day CPD course in biostatistics was designed, comprising of introductory, advanced and intermediate-level workshops. Conducted over three consecutive weekends, the workshops were designed to promote research output by providing HCPs with foundational skills for organizing and managing data using IBM-SPSS software, along with comprehensive knowledge and practical expertise in the analysis and interpretation of biostatistical data. Optional written assignments were designed to be completed following each workshop with the aim of fostering active learning and reinforcing the skills acquired during the sessions. A Certificate in the Analysis of Medical Data was awarded to participants who successfully completed all three assignments. The inclusion of a certificate was informed by recurring feedback from participants across various CPD programs at our institution, where certification was frequently identified as a desirable feature. It was therefore hoped that offering a certificate would enhance participant motivation and engagement in the program.

The three sets of workshops were delivered face-to-face during two cohorts, the first in 2019 and the second in 2020 and were open to all HCPs.

The workshops were designed to provide comprehensive training in biostatistical methods, as outlined in Table 1, which lists the specific statistical tests and objectives covered.

**Table 1:** Number and percentage of participants’ agreement with the extent to which each workshop learning objective was met*

	Learning Objective	Strongly agree *N* (%)	Agree *N* (%)	Neutral *N* (%)	Disagree *N* (%)	Strongly disagree *N* (%)
Introductory *N* = 89	Use IMB statistics to enter code and manage data	55 (62)	34 (38)	--	--	--
	Summarize variables in both numbers and graphs; and	56 (63)	33 (37)	--	--	--
	Use IBM-SPSS to apply basic analysis of numeric outcomes and categorical outcomes	54 (61)	33 (38)	2 (2)	--	--
Intermediate *N* = 75	Fit a linear regression to examine the relationship between a numeric dependent variable and one or more independent variables	44 (59)	25 (33)	6 (8)	--	--
	Fit a logistic regression to examine the relationship between a categorical dependent variable and one or more independent variables	46 (61)	25 (33)	4 (5)	--	--
	Test for interaction in regression;	43 (57)	26 (35)	6 (8)	--	--
	Assess confounding in regression.	41 (55)	28 (38)	6 (8)	--	--
Advanced *N* = 52	Generate a multiple linear regression	33 (63)	17 (33)	2 (4)	--	--
	Generate multiple logistic regression	30 (58)	20 (38)	2 (4)	--	--
	Analyze data from a one-way ANOVA	29 (56)	20 (38)	3 (6)	--	--
	Analyze data using non-parametric statistics	28 (54)	19 (36)	4 (8)	1 (2)	--
	Fit a Kaplan Meier curve and compute median survival	26 (50)	17 (33)	7 (13)	2 (4)	--
	Interpret Hazard ratios and their confidence intervals	25 (48)	18 (35)	8 (15)	1 (2)	--

* This Table presents self-reported agreement levels (Strongly Agree to Strongly Disagree) across specific learning objectives for each workshop level: Introductory (*N* = 89), Intermediate (*N* = 75), and Advanced (*N* = 52). Data are limited to participants who completed the post-activity evaluation survey. Two attendees from the Introductory workshop (total *N* = 91) did not submit responses, resulting in *N* = 89 for that category

While the intermediate and advanced workshops each addressed multiple statistical topics, the sessions were intended as introductory overviews rather than in-depth technical training. Each topic—for example, linear regression, logistic regression, and confounding—was covered at a conceptual level, with an emphasis on practical understanding and relevance to clinical research. The workshops incorporated real-world examples and software demonstrations to illustrate core principles, focusing on interpretation of results and common applications. Given the constraints of a one-day format, the content was carefully selected to provide a broad yet accessible foundation, suitable for participants with varying levels of prior experience. Supplementary materials, including all presentation slides and datasets for hands-on practice, were provided to support continued learning beyond the workshop. Participants who opted to pursue a certificate submitted post-workshop assignments and received detailed, individualized feedback. In cases where errors were identified, participants were encouraged to revise and resubmit their work. This iterative feedback process reinforced key learning objectives, particularly in selecting appropriate statistical methods, executing analyses using software, and interpreting results accurately.

Each workshop level—introductory, intermediate, and advanced—was structured to progressively build participants’ skills and understanding of biostatistics. Participants had the flexibility to register for individual workshops based on their specific needs and interests, rather than being required to attend all three sessions.

Participants engaged in hands-on experience with each statistical test listed in Table 1. The course tutor provided case studies and mock datasets to participants prior to the sessions. These materials were carefully crafted to simulate real-world research scenarios, allowing participants to apply the biostatistical methods in a practical context.

During the workshops, participants used IBM-SPSS software to practice each biostatistical method. This included performing data analysis, interpreting results, and understanding the application of statistical tests in healthcare research. The hands-on activities were integral to reinforcing theoretical knowledge and enhancing participants’ competence in using statistical software for data analysis.

By integrating case studies and practical exercises, the workshops aimed to equip participants with the skills necessary to conduct robust statistical analyses and apply these techniques in their professional practice.

Approximately one week post completion of each workshop, participants received the optional assignment. Assignments were completed using IBM-SPSS software in addition to written questions evaluating interpretation and understanding. Coursework underwent assessment by the course director and participants who successfully completed all three assignments were awarded with the certificate. The course was accredited by the Accreditation Council for Continuing Medical Education (ACCME).

### Design and data collection

A cross-sectional descriptive analysis utilized anonymous routine data collected during course administration and program evaluation. This involved a post-activity survey distributed upon completion of all accredited CPD activities. To address longitudinal objectives, including enhanced research output, an additional survey was administered 12-months following the final workshop. This extended timeframe aimed to ensure a thorough evaluation of the program’s impact on research productivity and provided participants with sufficient time for reflection on encountered barriers and challenges. The survey items were developed in line with ACCME accreditation standards, which are underpinned by Kirkpatrick’s Four-Level Training Evaluation Model [24]. Accordingly, the surveys assessed participant satisfaction with the workshop (Level 1), self-reported knowledge and competence (Level 2), and perceived application of statistical skills in research and practice (Level 3). Additionally, operational data from the CPD division included attendee numbers and numbers of participant certificates issued. Participation in the evaluation survey was voluntary and anonymous, and by completing the survey, participants provided implicit consent. Due to the anonymous nature of the post-activity surveys, individual progression through the workshops could not be tracked.

### Post-activity evaluation

One week following each workshop, participants received an email a link to the post-activity evaluation for accredited CE/CPD activities. The survey was designed to assess whether course objectives were met and is a standard survey sent to all participants completing CPD courses at WCM-Q (available in the supplementary file). The evaluation distinguished between multiple domains of impact, including perceived knowledge acquisition, competence, and performance, through separate survey items. These domains align with standard CPD outcome levels and were analysed individually. The survey consisted of single-item questions and was not designed for psychometric validation; therefore, internal consistency measures such as Cronbach’s alpha were not applicable. Completion was incentivized by offering a certificate of completion for each workshop.

The survey covered aspects such as registration, marketing, venue, speakers, and disclosure of commercial bias. This paper focuses on responses regarding the course’s perceived impact and achievement of objectives, rated on a 5-point Likert scale from strongly disagree to strongly agree. Questions included: 1) New knowledge acquisition, 2) Impact on competence, 3) Impact on performance, and 4) Potential effect on patient outcomes. Additionally, participants rated the workshop format on a 3-point Likert-scale (Yes, somewhat, no), with an open-ended question for further comments. The post-activity evaluation survey is a standard evaluation tool sent to all participants and was designed based on accreditation criteria (see Supplementary File).

### Program evaluation

In February 2021, an anonymous survey was emailed to all attendees of the 2019 and 2020 workshops using the Qualtrics survey distribution tool. This survey was designed to assess the specific objectives of the course and consisted of single-item Likert-scale and open-ended questions aligned with the course’s educational objectives (available in the supplementary file). As the survey was designed for descriptive evaluation rather than psychometric testing, internal reliability analysis (e.g., Cronbach’s alpha) was not conducted.

The survey aimed to evaluate impact of the workshops in supporting participants to draft and publish a manuscript as well as to understand the interpretation of data and perform statistical analyses (Fig. 2). Responses to 7 items (Fig. 2) were rated on a 5-point Likert scale. The survey also asked participants to confirm their certificate completion status, specify the sessions they attended, and provide reasons for not completing the program. Three open ended questions were also included to gauge potential difficulties participants encountered with completing the ‘Certificate in the Analysis of Medical Data’ as well as barriers towards achieving the long-term outcomes, and an opportunity for further comments.

Each participant completed the follow-up survey only once, regardless of how many workshops they attended. As such, Fig. 2 reflects unique individual responses rather than pooled data from multiple sessions.

The 2019 and 2020 cohorts were selected as they represent the first two fully completed cycles of the CPD biostatistics course. Evaluating these cohorts allowed for a structured assessment of the program’s immediate and long-term impact. Furthermore, the 2021 follow-up survey provided a sufficient post-course timeframe to assess changes in research productivity and application of biostatistical skills in practice.

A flowchart summarizing data collection and ass assessment in available in Supplementary Fig. 1.

### Analysis

Data from the surveys were summarized using frequencies and percentages using IBM-SPSS software (version 20, Armonk NY, USA). Due to the anonymous nature of the two surveys, it was not possible to cross-link responses between them. Open ended questions were categorised thematically and presented as frequencies of occurrence.

Due to the descriptive nature of this study, no formal sample size calculation was performed. All eligible participants from the 2019 and 2020 cohorts were invited to participate in the evaluation surveys. Response rates were calculated by dividing the number of completed surveys by the total number of invited participants and expressing this as a percentage.

The project received ethical exemption from the local Institutional Review Board (IRB reference number: 24 − 00022), as it involved routine program evaluation in an educational setting and did not involve collection of identifiable data.

## Results

The overall number of attendees at the introductory, intermediate, and advanced workshops for the 2019 and 2020 cohorts was 91, 84 and 60 respectively In 2019, the workshops were attended by 35 participants at the introductory level, 36 at the intermediate level, and 27 at the advanced level. In 2020, attendance was recorded as 56 for the introductory workshop, 48 for the intermediate, and 33 for the advanced. In 2019 administration data showed 11 participants (31% of those who initially registered) chose to complete the certificate of analysis of medical data. Fifteen participants (27% of those who initially registered) completed the certificate in 2020. A flow diagram summarizing workshop attendance across the 2019 and 2020 cohorts is available in Supplementary Fig. 1. Of the 235 attendees from both cohorts who received the link to the program evaluation survey, 216 (92%) completed the post-activity evaluation and 46 (20%) completed the program evaluation survey.

### Post-activity evaluation

The number and percentage of attendees completing the post-activity evaluation for the introductory, intermediate, and advanced workshops was 89 (98%), 75 (89%) and 52 (87%). Table 1 summarizes respondents’ levels of agreement regarding the attainment of the workshop objectives. Most responses exhibited near-unanimous agreement, ranging between 83% and 100%, encompassing individuals who both agreed and strongly agreed.

The level of agreement showed a decline for intermediate and advanced topics, as a notable number of participants expressed neutrality or disagreement regarding the achievement of objectives (Table 1). This trend was particularly noted in areas such as non-parametric statistics (10%), fitting a Kaplan Meier curve (17%), computing median survival, and interpreting Hazard ratios and their confidence intervals (17%).

**Fig. 1:**
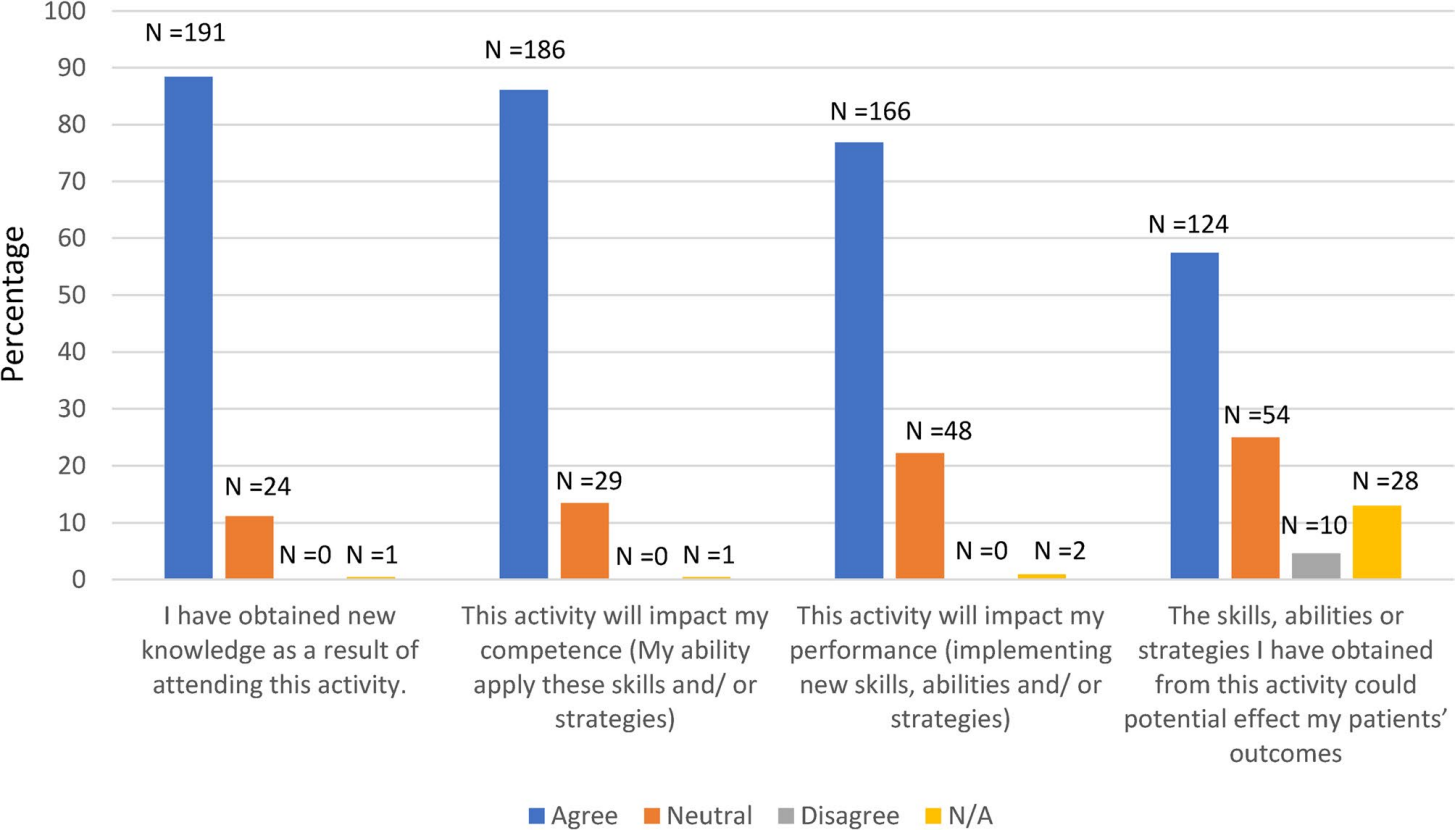
Perceived impact of training on knowledge, competence, performance and patient care. Responses to post-activity evaluation survey items (*N* = 216). Bars represent the percentage of participants selecting each response option (Agree, Neutral, Disagree, or N/A). N-values above each bar indicate the number of participants who responded to that specific item. All items reflect self-reported perceptions of impact on knowledge gained, competence (ability to apply skills), performance (implementation of new strategies), and perceived patient care outcomes. Percentages are based on item-level response counts

Findings from the post-activity evaluation are summarized in Fig. 1. The greatest perceived impact of the workshops was observed for obtaining new knowledge (88%) followed by impact on competence (86%) and performance (77%). Fewer participants felt that the skills obtained from the workshops could potentially affect patient outcomes (57%).

For all 216 participants completing the surveys, 209 (97%) agreed that the format was appropriate for the workshops, 6 (3%) felt it was somewhat appropriate and 1 (0.5%) did not agree.

### Qualitative insights

The open-ended question regarding the workshop format received 13 responses. Of these 5 respondents emphasized the need for a longer duration preferably over an additional day and including more time for questions and answers. Three respondents requested further case-based presentations, and three asked for more hands-on learning activities. One participant emphasized the necessity for greater participant engagement and further proposed the incorporation of breakout sessions for specific subtopics.

### Program evaluation

Of the forty-six respondents, 39 (85%) completed all workshop levels and 42 (91%) completed the introductory and intermediate workshops.

**Fig. 2:**
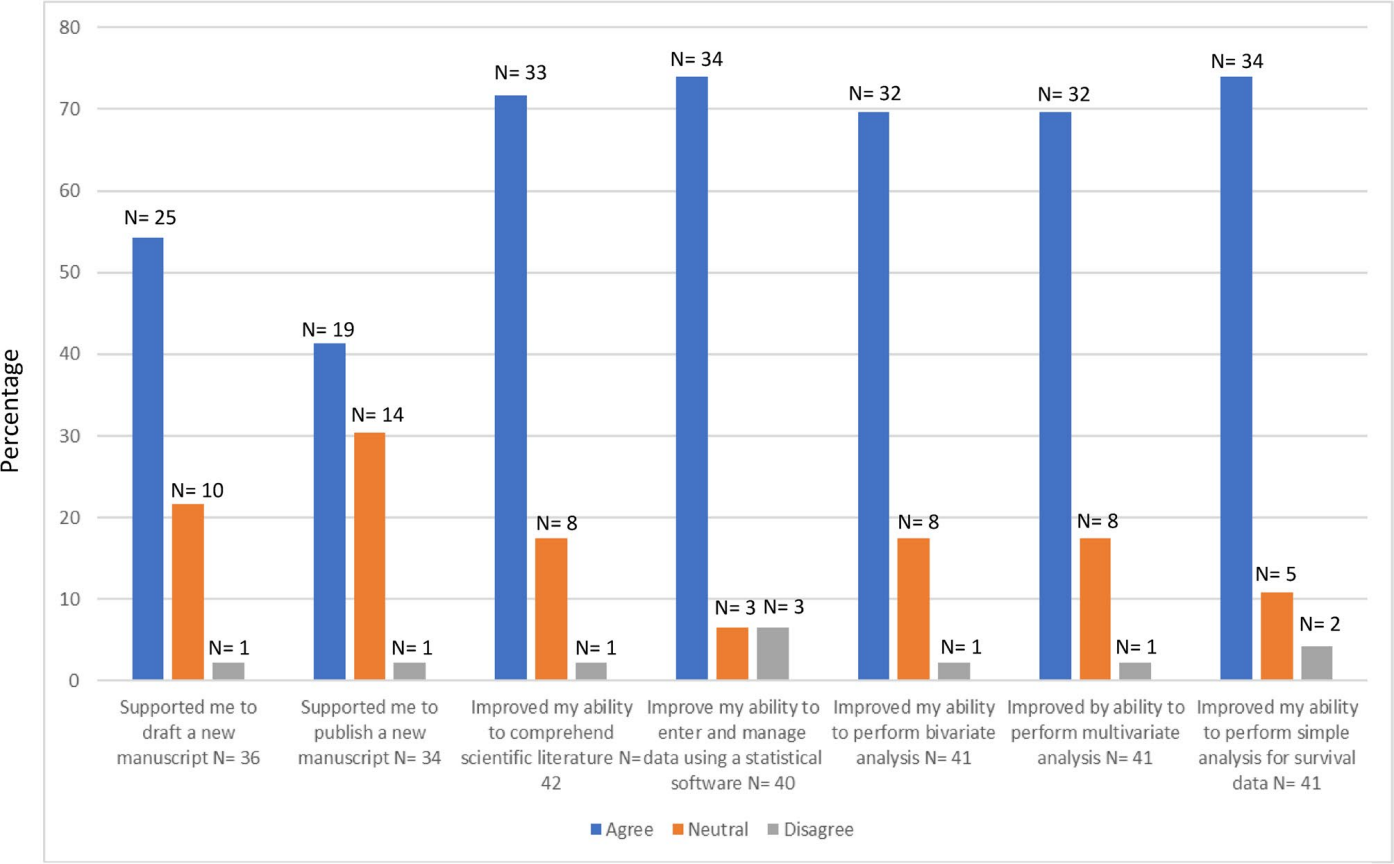
Perceived impact of training in supporting publication and ability to conduct statistical analysis. Perceived long-term impact of training on research engagement and statistical analysis. Data are based on responses from participants (*N* = 46) who completed a single follow-up survey conducted 12 months after the workshops. Each item shows the number of respondents (N) per response category. As each participant completed the survey only once, the data reflect unique individual responses. Percentages are based on the total number of responses per item

Participants’ perceived impact from attending the workshops is summarized in Fig. 2. Most participants felt that attending the workshops allowed them to improve their ability to enter and manage data on IBM-SPSS (74%) and conduct simple survival analysis (74%), as well as improve their understanding of scientific literature (72%). In comparison 54% felt the workshops had provided skills to enable them to draft a new manuscript and 41% credited the training with having provided support to publish a manuscript.

Twelve respondents answered the question “If you did not complete the certificate, could you please explain why you did not do so?”. Of these, three respondents discontinued the course following the introductory workshop and cited either not being able to attend in person: not being able to keep up with the pace of the session or the cost of the workshop as “*barriers”*. Two participants who discontinued the course following the intermediate class also cited not being able to attend in person. Six of the participants who provided a reason for not completing the certificate completed all three workshops. Reasons for not having completed the certificate included lack of time (*N* = 2), not being able to recall the material, having missed some of the course content, not needing the certificate and not having access to IBM-SPSS.

### Qualitative insights

Ten participants responded to the open-ended question regarding the barriers faced when considering the impact of the workshops as well as any other comments. In terms of barriers, five participants explained that they required more time to gain practical experience with the material they had covered during the sessions. Two participants requested either a recording of the workshops or them to be repeated online in order to review the material. One participant explained that they had not completed all the workshops to gain the necessary knowledge and skills to write a manuscript. Two participants highlighted the cost of the course as a potential barrier to future attendance.

## Discussion

Global interest towards enhanced research capacity among HCPs stems from numerous benefits including developing critical thinking skills and a culture of evidence-based practice which ultimately can improve practice and patient outcomes [1]. In line with this, Qatar has given significant priority to and made substantial investments in academic research, as outlined in QNV 2030 [8]. Nonetheless, for these investments to manifest as tangible research outcomes, it is imperative for HCPs to address obstacles that might impede research progress, including deficiencies in biostatistical knowledge.

This paper describes the process of design and the findings from an evaluation of a three-day CPD course in biostatistics for HCPs. The decision to develop the course as an accredited CPD activity ensured a structured and evidence-based methodology. Accreditation requirements also emphasized the importance of gathering and evaluating data to assess the aims and objectives, providing a robust framework to ensure the course’s relevance and impact. The evaluation design aligns with Kirkpatrick’s Four-Level Model, a widely used framework in CPD evaluation [24]. The instruments used in this study assessed outcomes at Levels 1 through 3—participant satisfaction, perceived knowledge and competence, and reported application of skills—providing a structured basis for interpreting participant feedback and guiding future course development.

The evaluation affirmed participants’ success in achieving course objectives, with some challenges identified in intermediate and advanced topics. Feedback revealed that while most participants found the workshop format acceptable, there was a preference for extended course duration and enhanced active learning opportunities. Additionally, participants expressed a preference for online and recorded materials to facilitate recall and comprehensive review. These findings align literature indicating extended course duration and ongoing exposure to material can yield improved outcomes and sustained changes [25]. Consequently, these findings underscore the importance of allocating sufficient time for in-depth exploration of advanced topics as well as a potential advantage in incorporating enduring material for future courses.

It is noteworthy that over two years only 29% of participants chose to complete the certificate, with physical attendance emerging as a significant barrier and time constraints and associated costs cited as reasons for not attending all three workshops. The literature consistently highlights time and cost as barriers to CPD attendance, prompting exploration of online modalities as a potential time-saving and cost-effective alternative [26, 27]. While research in Qatar indicates physicians’ openness to diverse CPD formats [26], additional studies are crucial to assessing participant experiences and comparing the effectiveness and cost-effectiveness of online delivery to traditional face-to-face workshops in teaching skills in biostatistics. Participants also highlighted a lack of necessity for the certificate, suggesting that that the incentive of a certificate may not be as effective as anticipated. This aligns with the findings from a recent study demonstrating HCP’s preferences for courses that enhance knowledge and skills, even without an associated qualification [28]. The findings therefore indicate a need to investigate alternative approaches including an emphasis on intrinsic motivation through activities that consolidate learning, to improve participant engagement with the material.

Participants reported high levels of perceived improvement in knowledge acquisition, competence, and performance. Notably, prior studies conducted among HCPs in Qatar identified low levels of research self-efficacy, particularly among pharmacists and physicians [6, 28]. Research self-efficacy refers to individuals’ confidence in applying research skills, including statistical analysis, which is essential for critically appraising published evidence and practicing evidence-based medicine [29]. Low self-efficacy in research has been associated with difficulties in clinical decision-making, reduced engagement in professional development, and lower research productivity [6, 29, 30]. In this context, the workshops may have helped enhance participants perceived ability to apply and integrate newly acquired skills. While the findings suggest a potentially positive step toward increased research engagement, further objective evidence is needed to confirm this impact. In addition, although the course also aimed to support evidence-based practice, system-level outcomes—such as improvements in patient care or institutional change—were not assessed in this evaluation.

Fewer HCPs perceived the course could affect patient outcomes compared to knowledge, competence, and performance, contrasting with previous research emphasizing positive attitudes towards research and evidence-based practice [7, 19, 31].

Nonetheless, improving patient outcomes remains a primary driver for promoting research-active practitioners [32]. Enhancing knowledge of statistical methods could enhance access to evidence-based medicine, potentially leading to better healthcare [17]. Further research is needed to understand factors influencing perceptions and potential interventions.

The longer-term program evaluation indicated that participants perceived continued benefits from the course, particularly in interpreting data and applying statistical analyses. Nearly half of the respondents also credited the course with supporting their ability to begin drafting or consider publishing a manuscript. While these findings are encouraging, they reflect the views of a subset of participants who completed the follow-up survey and may not fully capture the range of experiences across all attendees.

While highlighting the beneficial impact of a 3-day biostatistics course on HCPs’ perceived statistical analysis skills and research capacity, addressing limitations is essential. The anonymized nature of the data and the inability to correlate survey responses across various evaluation stages pose challenges in establishing causal relationships and exploring nuances between outcomes. It is essential to note that the program evaluation was not designed or powered to explore statistical differences; rather, it provides a descriptive snapshot of participant experiences.

Moreover, the risk of response bias and social desirability bias must be acknowledged, as self-reported survey data may lead participants to provide more favorable responses and potentially limit generalizability and inflating the perceived impact of the course. However, anonymity and framing the survey as a program evaluation rather than an individual assessment may have helped mitigate this effect. Self-selection bias is also a potential limitation, as participation in both surveys was voluntary. While the post-activity survey had a high response rate (92%), the follow-up survey response rate was lower (20%), introducing a risk of dropout bias. Due to the anonymous nature of data collection, it was not possible to compare respondents with non-respondents. Furthermore, this study did not compare knowledge gains between certificate completers and workshop attendees. Given that certificate completion was voluntary, differences in perceived learning may reflect self-selection bias.

While the long-term program evaluation demonstrated positive perceptions of the course’s perceived impact on participants’ ability to interpret data and perform statistical analyses, it is important to acknowledge that these insights are based on the responses of the 46 participants who completed the long-term evaluation survey. This represents a subset of the total participants, and as such, the findings may not fully capture the experiences and perceptions of all attendees. This limitation should be considered when interpreting the results, as it may affect the generalizability of the findings.

Although 42% of participants credited the course for supporting manuscript drafting, it was not possible to track actual publication rates due to the anonymous nature of the survey. Additionally, the timeline for manuscript development and publication often extends beyond the study’s 12-month follow-up period.

Future research should employ robust designs with objective measures including objective tracking of research output and longitudinal approaches, complemented by qualitative methods for more comprehensive understanding of the barriers and challenges faced by HCPs in acquiring statistical analysis skills and enhancing their research profile. Incorporating pre/post assessments or a control group would also facilitate evaluation of the impact of full certificate completion.

Our findings align with the broader goals of CPD programs in the Gulf Cooperation Council (GCC) region, which emphasize the importance of continuous professional development in healthcare. According to Alkhenizan & Shaw (2019), CPD programs are crucial for building healthcare capacity and should align with national health strategies [33]. The biostatistics continuing professional development course for HCPs in Qatar contributes to this objective by enhancing research skills and capacity among HCPs.

In line with our findings, international evaluations of similar CPD initiatives targeting research and evidence-based practice skills generally report improvements in knowledge and confidence but only modest changes in practice or research behavior. For example, Simons et al. (2019) found that evidence-based medicine training for physicians yielded short-term gains in knowledge and skills with little evidence of sustained changes in clinicians’ practice or attitudes [34]. Similarly, Tajuria et al. (2024) reported that a research capacity-building program in the UK increased participants’ self-rated research confidence and intentions, although lack of protected time and other barriers often impeded actual research engagement [35]. In a CPD workshop on critical appraisal skills for nurses, Tomotaki et al. (2024) observed a significant improvement in knowledge of research appraisal, yet most participants did not incorporate these skills into practice post-training (attributed to time constraints and workplace factors) [36]. Collectively, these studies underscore that while well-designed CPD courses can enhance healthcare professionals’ knowledge and perceived competence, measurable changes in performance or research output tend to be limited – a pattern that supports our cautious interpretation of the biostatistics course’s impact.

Internationally, CPD programs often utilize blended learning and self-paced modules to accommodate diverse learning preferences and schedules [37]. Subsequent to this study, course improvements, including extended duration and hybrid delivery options, have led to a rise in certificate completions. These findings offer insights into refining course structure and addressing challenges, contributing to ongoing research capacity development. However, the feasibility of fully transitioning to an online format requires careful consideration, particularly regarding engagement, hands-on statistical training, and technical support. Future iterations could explore a structured hybrid approach, integrating self-paced learning with interactive components to enhance accessibility while maintaining the practical nature of biostatistics education. As Qatar continues its investments in healthcare and research development, strengthening the research capabilities of HCPs becomes crucial for ensuring evidence-based practice. This evaluation suggests that the biostatistics course may have a positive perceived impact on participants’ knowledge, competence, and performance in statistical analysis. Although this course was designed to focus on biostatistical skill development, future training initiatives could incorporate a broader research methodology curriculum, including ethics, to further support responsible data analysis and reporting. Given the descriptive and self-reported nature of the findings, the results should be interpreted cautiously and seen as an initial step toward understanding the potential role of CPD in enhancing biostatistical competencies among HCPs.

## Conclusion

This study presents a tailored CPD course in biostatistics for Qatar’s HCPs, with findings suggesting a positive perceived impact on participants’ knowledge, competence, and performance in statistical analysis. Addressing biostatistical knowledge gaps through tailored CPD courses can enhance evidence-based practice and research productivity among HCPs. Insights from the study can inform the design of future CPD courses, potentially leading to improved healthcare outcomes and research productivity in Qatar and beyond. Further research is needed to assess the long-term outcomes of such training, including its impact on actual research output and clinical decision-making.

## Supplementary Information

Supplementary Material 1.

Supplementary Material 2.

Supplementary Material 3.

## Data Availability

The datasets used and/or analysed during the current study are available from the corresponding author on reasonable request.

## Abbreviations

HCP: Healthcare professionals
CPD: Continuing professional development
ACCME: Accreditation Council for Continuing Medical Education
