# Supplementary material for: Establishing a certificate in the analysis of medical data: a cross-sectional evaluation of a continuing professional development course in biostatistics in for healthcare professionals in Qatar
Source: BMC Med Educ. 2025 Oct 17;25:1435. doi: 10.1186/s12909-025-07999-7 (PMC12533324; doi:10.1186/s12909-025-07999-7)
Supplement: Supplementary file 1 — Supplementary Material 1. [file 12909_2025_7999_MOESM1_ESM.docx]

**Post-activity Evaluation**

Q1. I have obtained new knowledge as a result of attending this activity:

Strongly Agree

Agree

Neutral

Disagree

Strongly Disagree

Q.2 This activity will impact my competence (my ability to apply these new skills and/or strategies):

Strongly Agree

Agree

Neutral

Disagree

Strongly Disagree

Q3. This activity will impact my performance (implementing the new skills, abilities, and/or strategies into practice):

Strongly Agree

Agree

Neutral

Disagree

Strongly Disagree

Q4. The skills, abilities and/or strategies I have obtained from this activity could potentially affect my patients' outcomes:

Strongly Agree

Agree

Neutral

Disagree

Strongly Disagree

Q5. Were disclosures of speaker(s), moderator(s), facilitator(s) and/or author(s) clearly displayed for this activity?

Yes

No

Q6. Do you feel the activity had any commercial bias* or influence? *A commercial bias occurs when there is a current or potential financial interest with any entity Producing, Marketing, Re-Selling, or Distributing healthcare goods or services consumed by, or used on, Patients

No

Yes

Q7. Do you feel the activity was scientifically sound, evidence-based, objective, and balanced?

Yes

No

Q8. Was the format of this activity appropriate to the content presented?

Yes

No

Q9. OBJECTIVE - 1- Use IBM SPSS to enter, code and manage data

Strongly Agree

Agree

Neutral

Disagree

Strongly Disagree

Q10. OBJECTIVE - 2- Summarize variables both in numbers and graphs

Strongly Agree

Agree

Neutral

Disagree

Strongly Disagree

Q11. OBJECTIVE - 3- Use IBM SPSS to apply basic analysis of numeric outcomes and categorical outcomes

Strongly Agree

Agree

Neutral

Disagree

Strongly Disagree

Q12. The presenter was knowledgeable:

Strongly Agree

Agree

Neutral

Disagree

Strongly Disagree

Q13. The session met the stated presentation objectives:

Strongly Agree

Agree

Neutral

Disagree

Strongly Disagree

Q14. Overall, the session was beneficial: - Deema Al-Sheikhly, MRes, MEHP

Strongly Agree

Agree

Neutral

Disagree

Strongly Disagree

Q15. Based on your educational needs and/or perceived practice gaps in your specialty, please list any topics you would like to see addressed in future educational activities.

[free text-open ended question]

**Program Evaluation**

Q1 - Did you complete the Certificate in the Analysis of Medical Data: Applied Biostatistics for Healthcare Professionals?

Yes

No

Q2 - If you did not complete the certificate, which session(s) did you attend? (Please select all that apply)

Introductory

Intermediate

Advanced

Q3 - If you did not complete the certificate, could you please explain why you did not do so?

[Open ended text response]

Q4 - Would you be interested in attending a refresher course if it was offered every 2 years

Yes

No

Q5 - Please choose to what degree you agree with the following statements regarding the course The training provided has significantly:

[Reponses on a 5-point likert scale; strongly agree, agree, neutral, disagree and strongly disagree]

Supported me to draft a new manuscript

Supported me to publish a new manuscript

Improved my ability to comprehend scientific literature

Improved my ability to enter and manage data using a statistical software

Improved my ability to perform bivariate analysis for both continuous and dichotomous outcomes

Improved my ability to perform multivariate analysis for both continuous and dichotomous outcomes

Improved my ability to perform simple analysis for survival data

Q6 - If you selected Disagree or Strongly disagree to any of the above, please provide an explanation why and/or any barriers faced

[open ended free text response]

Q7 - Please add any other comments or feedback you may have

[open ended free-text response]
