# Supplementary material for: Establishing a certificate in the analysis of medical data: a cross-sectional evaluation of a continuing professional development course in biostatistics in for healthcare professionals in Qatar
Source: BMC Med Educ. 2025 Oct 17;25:1435. doi: 10.1186/s12909-025-07999-7 (PMC12533324; doi:10.1186/s12909-025-07999-7)
Supplement: Supplementary file 2 — Supplementary Material 2. [file 12909_2025_7999_MOESM2_ESM.docx]

Supplementary Figure 1. Flowchart of Workshop Attendance Across Introductory, Intermediate, and Advanced Levels (2019–2020 Cohorts) and Data Collection and Analysis Process. N = number of participants.

**Introduction**

**Intermediate**

**Advanced**

**2020 N**

N =56

N =48

N =33

**Total**

N = 91

N =84

N =60

**2019 N**

N =35

N =36

N =27

216 post-activity evaluations completed (2019 and 2020)

46 program evaluation surveys completed (2019 and 2020)

Data Collection

**Timeline**: 1 week post attendance

**Assessment**:

1. Course objectives (Number and percentage reported in Table. 1).

2. Perceived impact (Frequencies reported in Figure. 1).

**Flowchart of workshop attendance across the 2019–2020 cohorts.** The numbers represent the total number of participants per workshop, not repeated assessments. While the course was designed as a structured progression from introductory to advanced levels, participants had the flexibility to enrol in individual workshops based on their prior experience and learning needs. Although formal tracking data were not collected, course director feedback suggests that most participants followed the full sequence, with a few skipping sessions due to scheduling conflicts or existing knowledge. The arrows indicate the intended course structure rather than a mandatory progression

This diagram reflects total workshop attendance, whereas Table 1 reports only those who completed post-activity evaluations. For the introductory workshop, 91 participants attended, but only 89 completed the evaluation survey, accounting for the difference in numbers

**Timeline**: 12 months post final workshop

**Assessment**

1. Achievement of long-term objectives (Frequencies in Figure. 2)

2. Open ended questions. Categorized and frequencies reported in results
