# Supplementary material for: Establishing a certificate in the analysis of medical data: a cross-sectional evaluation of a continuing professional development course in biostatistics in for healthcare professionals in Qatar
Source: BMC Med Educ. 2025 Oct 17;25:1435. doi: 10.1186/s12909-025-07999-7 (PMC12533324; doi:10.1186/s12909-025-07999-7)
Supplement: Supplementary file 3 — Supplementary Material 3. [file 12909_2025_7999_MOESM3_ESM.docx]

**Supplementary file 2. Perceived impact of training on knowledge, competence, performance and patient care. Data presented by workshop per year**

Supplementary Table 1. Number and percentage of participants agreeing with each statement in 2019 and 2020: Introductory workshop. *

|  | I have obtained new knowledge as a result of attending this activity | | This activity will impact my competence (My ability to apply these skills and/ or strategies) | | This activity will impact my performance (implementing new skills, abilities and/ or strategies) | | The skills, abilities or strategies I have gained from this activity could potentially affect my patients’ outcomes | |
| --- | --- | --- | --- | --- | --- | --- | --- | --- |
|  | 2019 | 2000 | 2019 | 2000 | 2019 | 2000 | 2019 | 2000 |
| Agree | 32 (94.1) | 47 (85.5) | 31 (91.2) | 46 (83.6) | 30 (88.2) | 40 (72.7) | 21 (61.8) | 26 (47.3) |
| Disagree | -- | -- | -- | -- | -- | -- | -- | 1 (1.8) |
| Neutral | 2 (5.9) | 8 (14.5) | 3 (8.8) | 9 (16.4) | 4 (11.8) | 15 (27.3) | 8 (23.5) | 21 (38.2) |
| N/A | -- | -- | -- | -- | -- | -- | 5 (14.7) | 7 (12.7) |

* Number of responses per session: 2019 (N =34); 2020 (N =55).

Supplementary Table 2. Number and percentage of participants agreeing with each statement in 2019 and 2020: Intermediate workshop.*

|  | I have obtained new knowledge as a result of attending this activity | | This activity will impact my competence (My ability to apply these skills and/ or strategies) | | This activity will impact my performance (implementing new skills, abilities and/ or strategies) | | The skills, abilities or strategies I have gained from this activity could potentially affect my patients’ outcomes | |
| --- | --- | --- | --- | --- | --- | --- | --- | --- |
|  | 2019 | 2000 | 2019 | 2000 | 2019 | 2000 | 2019 | 2000 |
| Agree | 27 (87.1) | 40 (90.9) | 26 (83.9) | 39 (88.6) | 24 (77.4) | 33 (75) | 19 (61.3) | 24 (54.5) |
| Disagree | -- | -- | -- | -- | -- | -- | -- | 1 (2.3) |
| Neutral | 4 (12.9) | 3 (6.8) | 5 (16.1) | 4 (9.1) | 7 (22.6) | 9 (20.5) | 7 (22.6) | 8 (18.2) |
| N/A | -- | 1 (2.3) | -- | 1 (2.3) | -- | 2 (4.5) | 5 (16.1) | 11 (25) |

* Number of responses per session: 2019 (N =31); 2020 (N =44).

Supplementary Table 3. Number and percentage of participants agreeing with each statement in 2019 and 2020: Advanced workshop.

|  | I have obtained new knowledge as a result of attending this activity | | This activity will impact my competence (My ability to apply these skills and/ or strategies) | | This activity will impact my performance (implementing new skills, abilities and/ or strategies) | | The skills, abilities or strategies I have gained from this activity could potentially affect my patients’ outcomes | |
| --- | --- | --- | --- | --- | --- | --- | --- | --- |
|  | 2019 | 2000 | 2019 | 2000 | 2019 | 2000 | 2019 | 2000 |
| Agree | 20 (83.3) | 25 (89.3) | 18 (75) | 26 (92.9) | 16 (66.7) | 23 (82.1) | 19 (79.2) | 15 (53.6) |
| Disagree | -- | -- | -- | -- | -- | -- | -- | 1 (3.6) |
| Neutral | 4 (16.7) | 3 (10.7) | 6 (25) | 2 (7.1) | 8 (33.3) | 5 (17.9) | 4 (16.7) | 6 (21.4) |
| N/A | -- | -- | -- | -- | -- | -- | 1 (4.2) | 6 (21.4) |

* Number of responses per session: 2019 (N =24); 2020 (N =28).
